# Sugar sweetened beverage consumption is positively associated with Klotho levels at two years of age in LatinX youth

**DOI:** 10.1186/s40795-021-00423-5

**Published:** 2021-04-23

**Authors:** Sofia Villagomez, Dena B. Dubal, Jessica Hawkins, Dan Wang, Janet M. Wojcicki

**Affiliations:** 1 Department of Pediatrics, University of California, 550 16th Street, San Francisco, USA; 2 Department of Neurology, University of California, San Francisco, USA

**Keywords:** Klotho, Sugar sweetened beverages, LatinX youth

## Abstract

**Background:**

Klotho is an anti-aging protein mainly expressed in the kidneys with a smaller amount expressed in adipose tissue. Klotho effects include roles in reducing oxidative stress, insulin signaling, adipogenesis and glucose metabolism. Few studies have investigated the role of dietary factors such as sugar sweetened beverages (SSBs) on serum α-klotho levels in young children.

**Methods:**

Data was collected from 60 low-income Latina pregnant women and their infants in San Francisco from birth until 2 years of life and examined for associations between dietary factors and child secreted α-klotho protein levels at 2 years.

**Results:**

Mean α-klotho levels were 1782.96 ± 874.56 pg/mL at 2 years of age. Any consumption of SSBs was independently associated with increased α-klotho levels (Beta = 682.79, 95%CI 67.50, 1298.09; *p* = 0.03). Household income ranging from $25,000 to $50,000 was also correlated to higher levels of α-klotho in children compared with lower income levels (<$25,000) (Beta = 1613.35, 95%CI 527.37, 2699.33; *p* = 0.005).

**Conclusions:**

The positive association between SSB intake and α-klotho levels at 2 years may reflect higher phosphate levels consistent with SSB intake. Higher socioeconomic status may be a proxy for reduced stress exposure in children, also associated with higher α-klotho levels. Future studies should evaluate the early impact of exposures to SSBs, stress and accelerated aging in children.

## Background

Klotho is a protein existing in multiple forms (membrane bound, intracellular, and secreted or α-klotho, β-klotho, and γ-klotho), encoded by the Klotho gene [1]. α-Klotho is the form of the protein that plays a major role in ion homeostasis and interacts with fibroblast growth factor − 23 (FGF-23), a phosphaturic hormone derived from bone, that inhibits inorganic phosphate renal uptake [1].

The effects of α-klotho include roles in reducing oxidative stress, insulin signaling, adipogenesis, glucose and calcium metabolism [2]. Due to its multiple roles in homeostasis, metabolism and potentially chronic disease risk, certain α-klotho allele combinations are referred to as “anti-aging” genotypes. In mouse models, higher α-klotho expression is associated with a longer life [1] while in humans secreted α-klotho protein levels increased post exercise regimens [3] leading to the interpretation that it is potentially also a fitness marker in addition to a health marker. Considering the potential life-long benefits of maintaining, if not increasing secreted α-klotho in preventing disease and supporting metabolism it is important to understand the dietary, socioeconomic, and hormonal factors which could impact circulating levels of this important protein.

One of α-klotho’s key roles in the body is in mineral metabolism, especially phosphate and calcium [1]. Secreted α-klotho maintains ion homeostasis by inhibiting renal and intestinal phosphate (P_i_) transporters to decrease plasma phosphate concentrations [1]. α-Klotho and FGF-23 work in combination to increase urinary phosphorous and calcium reabsorption for optimal concentration of CA^2+^ and P_i_ in bone and blood [4]. In normal physiology, an increase in phosphate leads to an increase of both α-klotho and FGF-23, which suppresses IGF-1 signaling [5], an important growth hormone.

High levels of dietary phosphate have adverse consequences especially for those with impaired kidney function, but new understandings of hormonal mechanisms including FGF-23 and α-klotho [6] promotes a mechanism of potential harm [7] without compromised kidney function. It is possible that early exposures in life to higher phosphate levels may be more detrimental than later ones. A study in mice found that during early life, a diet high in phosphate was associated with a stronger adverse effect on α-klotho expression in the kidneys, than in mice fed the same diet during a later (peri-adolescent) growth period [7]. This is of concern for health in all populations due to the increased use of phosphate as a food additive, (e.g. processed meats, and sodas) which is now the most common dietary source of phosphate [8]. In the USA hyperphosphatemia (defined as an abnormally high serum phosphate concentration of > 4.4 mg/dL) [9] has been found to be twice as common among persons of low income than among persons of high income likely due to the high concentration of phosphate in low-cost food items [8].

The cohort described here represents a predominantly low-income population with potentially high sugar-sweetened beverage (SSB) intake. In this context, we assessed potential connections between diet and α-klotho levels in the first few years of life.

## Methods

In our previously described Latinx, Eating and Diabetes cohort (LEAD) *n* = 97 of pregnant, self-identified Latina women, cord blood was collected from a subset of women (*n* = 72) as were infant anthropometrics as previously described [10, 11]. At 2 years of age, during the time that many children have an additional blood draw to check for lead levels as part of the Special Supplemental Nutrition Program for Women, Infants and Children (WIC) program in California, children had an additional venous blood draw. Additional specifics of the study are described below.

### Socio-demographic and clinical data

Maternal socio-demographics including race, ethnicity, age, marital status and employment were collected at enrollment as previously described [10, 11]. Women were assessed for prenatal depression using the Edinburgh Postnatal Depression Scale (EPDS) and the Center for Epidemiological Depression Scale (CESD) in pregnancy [11]. Infant anthropometrics collected at birth using standard digital scales and tape measures and birth characteristics were extracted from the medical record (Apgar scores and gestational age) [11]. Infant anthropometrics (weight and length/height) were similarly assessed at 6 months, 12 months, and 2 years of age. Standard Center for Disease Control (CDC) growth curves were used to define childhood obesity as weight for length or body mass index (BMI) percentile ≥95th [11, 12]. Food frequency questionnaires assessed infant and toddler dietary intake at 4–6 weeks, 6 months, 1 and 2 years of age. Exclusive breastfeeding was defined as consumption of only breastmilk without the addition of any other foods or beverages including water using the World Health Organization (WHO) definition [13]. SSBs were defined as any beverage with added sugars included sodas, fruit drinks, sweetened milk beverages and sports drinks. High intake of 100% fruit juice consumption was defined as more than 4 servings per week and high intake of SSBs was defined as more than 4 servings per week. All women provided written consent for their own and their children’s participation as all children were below the age of written and verbal consent. The study was approved by the Institutional Review Board (IRB) of the University of California, San Francisco (Committee on Human Research (CHR)).

### Laboratory

Briefly, serum leptin, insulin, insulin-like growth factor 1 (IGF-1) were assayed (Milliplex MAP Human Metabolic Hormone Magnetic Bead Panel [Millipore], Linco, St Charles, MO, USA). The minimum detectable concentration for leptin was 27 pg/mL and insulin 58 pg/mL. The intra-assay coefficient of variation (CV) and inter-assay CV for leptin and insulin were 3 and 4% respectively. Serum IGF-1 was assessed using the Milliplex MAP Human IGF-1 Single Plex. The minimum detectable concentration was 52 pg/mL and the intra-assay CV was 4% and the inter-assay was 7%.

Serum α-klotho levels were measured in plasma using a solid-phase sandwich enzyme-linked immunosorbent assay (Immuno-Biological Laboratories, Takasaki, Japan) by the Dubal lab at UCSF as previously described [11]. All samples were run in duplicate. The assay had an average intra-assay CV of approximately 3–5%. Samples with a CV above 7% were repeated with 16 samples being re-run and the control sample in the second run being in close agreement with the first run.

### Statistical methods

α-Klotho values at 2 years of age, as the primary outcome of interest, were assessed for normal distribution through graphing and tests for normality including Shapiro-Wilk and Shapiro-Francia tests [11]. As the data was not normally distributed, we used non-parametric tests of association. Wilcoxon rank sum (Mann-Whitney) test was used for dichotomous predictors of α-klotho levels and Kruskal-Wallis for categorical predictors [11]. Association between α-klotho levels and continuous predictors were assessed with Spearman’s rank correlation coefficients (ρ). Statistical significance was determined at *P*-values < 0.05. Data in tables are expressed as means plus or minus standard deviations and percentages [11]. We also assessed normality for linear regression models. Linear regression residuals were not normally distributed. We tested a bootstrapped sample with 1000 replications and compared findings to regression coefficients which were not bootstrapped. As there were no differences in regression results between the bootstrapped and non-bootstrapped finding, we assumed that departures from normality were related to sample size and not to other factors. As such, we used linear regressions to test associations.

Multivariable analyses included those variables that were significant at *p* < 0.20, a threshold chosen to reduce the possibility of Type II errors, in addition to those variables that previously demonstrated a plausible association with α-klotho levels including insulin and leptin levels. We did not include variables that had a small cell size < 5 in bivariate analysis in our multivariable models. Correlation tests were run on all variables and any which showed R > 0.7 were not included simultaneously in models. Beta coefficients are presented with 95% confidence intervals (CI) and *p*-values. Robust multivariable regression models were subsequently compared with initial models to assess for the possible influence of outliers.

## Results

Of the 97 parent/child dyads included in the LEAD cohort, secreted α-klotho protein measurements taken at 2 years were available for 60 (62% of the sample) children. Mean α-klotho levels were 1783.0 ± 874.6 pg/mL at 2 years of age. There were few differences between families who had child α-klotho measured at two years of age and those who did not including maternal age (*p* = 0.09), child birthweight percentile (*p* = 0.75), family income (*p* = 0.93), and maternal marital status (*p* = 0.44). Families who did have α-klotho measured were more likely to be Mexican versus Central or South American origin (*p* < 0.01) and have higher maternal education levels (*p* = 0.03).

### Maternal characteristics

Increasing maternal education level was associated with higher α-klotho levels with children of high school graduates having 2359.83 ± 1387.12 pg/mL compared to 1690.28 ± 755.59 pg/mL (*p* = 0.045) for the children of mothers with less than a high school education (Table 1). There was no association between other maternal demographic characteristics and α-klotho levels including maternal age or health during/prior to pregnancy including pre-pregnancy BMI, or weight gain in pregnancy (Table 1). Self-reported family income levels 25 K–50 K at time of enrollment neared statistical significance for higher child levels of α-klotho compared to family income of < 25 K (2377.70 ± 1758.81 pg/mL versus 1716.88 ± 717.72 pg/mL) (*p* = 0.079; Table 1).

**Table 1:** Maternal Characteristics and α-Klotho Levels at 2 Years

Variable	Population mean ± Standard Deviation or n/total	α-Klotho levels at 2 years mean ± standard deviation, pg/mL	***p***-value
**Health Characteristics**			
Pre-Pregnancy body mass index (BMI), kg/m2	27.52 ± 4.62	1789.42 ± 898.99	0.83
Underweight or Normal	12/55 (22)	1852.09 ± 1214.14	
Overweight	22/55 (40)	1682.67 ± 710.73	
Obese	21/55 (38)	1865.44 ± 905.52	
Excess weight gain in pregnancy			0.18
No	27/54 (50)	1591.09 ± 465.31	
Yes	27/54 (50)	1906.52 ± 1109.41	
Depressive symptoms in pregnancy			0.79
No	48/60 (80)	1798.40 ± 894.30	
Yes	12/60 (20)	1721.19 ± 824.66	
Age of mom at time of delivery	28.41 ± 5.34	1782.96 ± 874.56	0.74
< 35 years	50/60 (83)	1819.18 ± 919.34	
> =35 years	10/60 (17)	1601.85 ± 607.32	
**Socio-Demographics**			
Maternal Education			
Less than high school	51/59 (86)	1690.28 ± 755.59	
High school graduate or more	8/59 (14)	2359.83 ± 1387.12	0.05
Marital Status			
Single or divorced/eparated/widowed	9/60 (15)	1565.23 ± 876.99	
Living with partner or married	51/60 (85)	1821.37 ± 877.18	0.42
Self-Reported Household Income			
Less than 25,000	54/60 (90)	1716.88 ± 717.72	
25,000-50,000	6/60 (10)	2377.70 ± 1758.81	0.08

### Child characteristics

We did not find any association between birth characteristics including child sex, gestational age, Apgar score at 5 min and α-klotho levels. However, birthweight percentile below the 50th percentile neared statistical significance for increased α-klotho levels compared with birthweight percentile greater or equal to the 50th percentile (2133.42 ± 1079.41 pg/mL versus 1448.03 ± 283.36 pg/mL *p* = 0.055; Table 2). Other birth anthropometrics including being macrosomic (> 4000 g), underweight (< 2500 g) or preterm (< 37 weeks gestational age) were not associated with α-klotho levels at 2 years of age. Similarly, head circumference percentile and obesity in early childhood (6 months, 12 months, 24 months) were not associated with α-klotho levels at 2 years of age. We also did not find any association between infant and toddler adiposity or growth hormone levels taken at birth or two years (IGF-1, ghrelin, insulin, leptin) and α-klotho levels at 2 years. Male children tended to have higher α-klotho levels (Beta = − 396.21, 95%CI -1064.77-272.35; *p* = 0.24) but the association was not statistically significant (Table 2).

**Table 2:** Child Characteristics and α-Klotho Levels at 2 Years of Age

Variable	Population mean ± Standard Deviation or n/total	α-Klotho levels at 2 years mean ± standard deviation, pg/mL	***p***-value
**Birth Characteristics**			
Child sex			
Female	30/60 (50)	1900.78 ± 860.19	0.30
Male	30/60 (50)	1665.14 ± 887.38	
Gestational age (weeks)	39.12 ± 1.37	1782.96 ± 874.56	0.25
< 40	44/60 (73)	1735.59 ± 828.01	
> = 40	16/60 (27)	1913.22 ± 1009.15	
Gestational age (tertiles)	39.12 ± 1.37	1782.96 ± 874.56	0.25
< 39	23/60 (39)	1653.50 ± 925.44	
39–39.8	20/60 (33)	1852.85 ± 725.43	
> 39.8	17/60 (28)	1875.75 ± 989.24	
Apgar score at 5 min	8.8 ± 0.6	1782.96 ± 874.56	0.55
< 9	11/60 (18)	1527.74 ± 448.58	
> = 9	49/60 (82)	1840.25 ± − 938.05	
**Birth Anthropometrics**			
Birth weight, g	3384.09 ± 471.41	1782.96 ± 874.56	0.41
Macrosomic at birth (> 4000 g)			
No	57/60 (95)	1779.65 ± 891.88	0.90
Yes	3/60 (5)	1845.77 ± 533.61	
Underweight (< 2500 g) or preterm (< 37 weeks)			
No	54/60 (90)	1820.17 ± 910.91	0.33
Yes	6/60 (10)	1448.03 ± 283.36	
Birth weight percentile	56.53 +/− 25.40	1782.96 ± 874.56	0.05
< 50%	24/60 (40)	2133.42 ± 1079 .41	0.01
> = 50%	36/60 (60)	1549.31 ± 619.12	
Birth weight for length below 5th % ile			
No	51/60 (85)	1846.64 ± 919.01	0.18
Yes	9/60 (15)	1422.09 ± 435.82	
Head circumference percentile (tertiles)	34.20 +/− 1.42	1782.96 ± 874.56	0.38
< 25%	36/60 (60)	1511.39 ± 543.04	
25–50%	18/60 (30)	2398.09 ± 1199.40	
> 50%	6/60 (10)	1566.93 ± 347.65	
**Early childhood anthropometrics**			
Obesity in early childhood			
6 months (Yes)	7/60 (12)	1681.31 ± 685.04	0.75
No	53/60 (88)	1796.38 ± 901.17	
12 months (Yes)	7/60 (12)	1656.52 ± 761.34	0.69
No	53/60 (88)	1799.65 ± 893.59	
24 months (Yes)	6/60 (10)	1975.48 ± 790.31	0.57
No	52/60 (90)	1756.77 ± 888.70	
**Hormone Levels**			
IGF-1 at birth (pg/mL)	30,749.25 ± 21,214.53	1792.06.39 ± 953.16	0.71
< 17,600	14/46 (31)	1559.26 ± 682.81	0.49
17,600–35,120	17/46 (40)	1814.85 ± 901.79	
> 35,120	15/46 (33)	1983.53 ± 1209.86	
IGF-1 at 2 years	29,126.19 ± 15,992.23	1782.96 ± 874.56	0.42
< 17,600	18/60 (30)	1597.60 ± 663.41	0.54
17,600–35,120	27/60 (45)	1893.88 ± 1020.88	
> 35,120	15/60 (25)	1805.73 ± 827.61	
Ghrelin at birth (pg/mL)	39 ± 22	1499.55 ± 367.95	0.40
< 22	4/13 (31)	1544.74 ± 563.03	0.85
22–45	7/13 (54)	1444.72 ± 268.62	
> 45	2/13 (15)	1601.06 ± 426. 68	
Insulin at birth (pg/mL)	953.76 ± 546.78	1801.79 ± 985.83	0.18
> 750	17/41 (41)	1823.06 ± 1112.10	0.65
750–1060	16/41 (39)	1920.01 ± 986.86	
> 1060	8/41 (20)	1520.18 ± 718.30	
Insulin at 2 years (pg/mL)	766.17 ± 900.33	1782.96 ± 874.56	0.35
> 750	41/60 (68)	1706.81 ± 888.29	0.60
750–1060	8/60 (13)	1895.41 ± 736.32	
> 1060	11/60 (18)	1984.96 ± 947.67	
Leptin at birth (pg/mL)	23,025.87 ± 18,375.04	1766.25 ± 989.08	0.82
< 11,200	12/39 (30)	1827.42 ± 984.01	0.70
11,200–27,000	17/39 (44)	1618.32 ± 1063.94	
> 27,000	10/39 (26)	1944.32 ± 923.65	
Leptin at 2 years (pg/mL)	733.93 ± 1267.03	1782.96 ± 874.56	0.85
< 3000	58/60 (96)	1789.65 ± 884.72	0.72
3000–6000	1/60 (2)	1123.12	
> 8000	1/60 (2)	2054.59	

### Child dietary factors

There was also no association between breastfeeding (exclusive or any), any formula intake, 100% juice intake and α-klotho levels at 2 years of age. Exclusive breastfeeding trended towards statistical significance with higher α-klotho levels among those infants being exclusively breastfed at six months (defined by WHO as: “no other food or drink, not even water, except breast milk for 6 months of life, but allows the infant to receive ORS, drops and syrups [13]”) versus those that were not (2561.18 ± 1384.99 pg/mL versus 1754.47 ± 851.26 pg/mL; *p* = 0.126; Table 3). At 24 months of age, any SSB consumption was associated with higher α-klotho levels (2029.78 ± 999.45 versus 1402.78 ± 496.28; *p* < 0.01; Table 3).

**Table 3:** Child Dietary Intake and α-Klotho Levels at 2 Years

Variable	Population mean +/− Standard Deviation or n/total	α-Klotho levels at 2 years mean +/− standard deviation, pg/mL	***p***-value
**Breastfeeding**			
Any			
4–6 weeks (Yes)	59/60 (98)	1795.15 ± 876.92	0.70
No	1/60 (2)	1063.83	
6 months (Yes)	42/58 (72)	1825.55 ± 900.11	0.69
No	16/58 (28)	1719.15 ± 874.44	
12 months (Yes)	36/59 (61)	1759.55 ± 756.31	0.70
No	23/59 (39)	1850.87 ± 1054.50	
Exclusive			
6 months (Yes)	3/58 (5)	2561.18 ± 1384.99	0.13
No	55/58 (95)	1754.47 ± 851.26	
12 months (Yes)	20/60 (33)	1875.99 ± 850.63	0.56
No	40/60 (66)	1736.44 ± 893.27	
**Infant Formula**			
4–6 weeks (Yes)	31/60 (52)	1717.74 ± 933.12	0.55
No	29/60 (48)	1852.66 ± 817.94	
**100% Fruit Juice**			
Any			
6 months (yes)	17/57 (30)	1608.98 ± 694.53	0.30
No	40/57 (70)	1878.06 ± 963.75	
12 months (yes)	46/59 (78)	1769.26 ± 883.68	0.67
No	13/59 (22)	1886.74 ± 881.32	
24 months (yes)	55/56 (98)	1803.69 ± 890.42	0.28
No	1/56 (2)	832.76	
High Intake			
24 months (yes)	18/56 (32)	1681.84 ± 669.55	0.55
No	38/56 (68)	1835.85 ± 984.04	
**Sugar Sweetened Beverages**			
Any			
12 months (yes)	17/59 (29)	1631.34 ± 584.62	0.37
No	42/59 (71)	1861.45 ± 968.93	
24 months (yes)	34/58 (59)	2029.78 ± 999.45	0.01
No	24/58 (41)	1402.78 ± 496.28	
High Intake			
24 months (yes)	9/58 (16)	1603.39 ± 859.46	0.54
No	49/58 (84)	1800.99 ± 889.21	

### Multivariable analysis

Adjusting for weight z-score at birth, leptin levels at 2 years, insulin levels at birth, maternal education level, household income, and child sex, any consumption of SSBs at 24 months was associated with increased α-klotho levels in children (Beta = 682.79, 95%CI 67.50, 1298.09.1; *p* = 0.03) (Table 4). Household income ranging from $25,000 to $50,000 per year was also significantly associated with higher levels of childhood α-klotho levels compared to those with lower income  levels (Beta = 1613.35, 95%CI 527.37, 2699.33; *p* = 0.005) (Table 4). When insulin was excluded from our final multivariable regression to provide more power and a larger sample size, SSB intake approached significance (Beta = 434.68, 95%CI -20.33, 889.68; *p* = 0.06) as did higher maternal education level (Beta = 647.57; 95%CI -53.41-1348.55; *p* = 0.07) and higher household income (Beta = 696.78; 95%CI -69.28-1462.85 *p* = 0.074) (Table 5).

**Table 4:** Final Multivariable Model Summary

			Number of Observations =			39
				Probability > F =		0.0119
				R-Squared =		0.4177
				Adjusted R-Squared =		0.2862
				Root MSE =		853.8
α-Klotho Levels at 2 years	Coefficient	Standard Error	t	*P* > |t|	[95% Conf. Interval]	
Weight Z Score at Birth	−17.15	219.30	−0.08	0.94	− 464.41	430.11
Leptin at 2 Years	−0.04	0.09	−0.41	0.68	−0.23	0.15
Insulin at Birth	−0.14	0.35	−0.41	0.68	−0.85	0.56
SSB Intake at 2 years (yes)	682.79	301.69	2.26	0.03	67.50	1298.09
Child Sex (male)	−396.21	327.80	−1.21	0.24	− 1064.77	272.35
Maternal Education (high school graduate or more)	373.68	449.38	0.83	0.41	− 542.83	1290.20
Household Income (25 k–50 k)	1613.35	532.47	3.03	0.005	527.37	2699.33

**Table 5:** Final Multivariable Model Summary, Excluding Insulin at Birth

			Number of Observations =			57
				Probability > F =		0.0121
				R-Squared =		0.2698
				Root MSE =		802.94
α-Klotho levels at 2 years	Coefficient	Standard Error	t	P > |t|	[95% Conf. Interval]	
Weight Z Score at Birth	− 143.87	133.16	−1.08	0.28	−411.34	123.59
Leptin at 2 Years	−0.06	0.08	−0.65	0.52	−0.23	0.12
SSB Intake at 2 years (yes)	434.68	226.53	1.92	0.06	−20.33	889.68
Child Sex (male)	− 376.17	227.69	−1.65	0.10	− 833.51	81.17
Maternal Education (high school graduate or more)	647.57	348.99	1.86	0.07	−53.41	1348.55
Household Income (25 k–50 k)	696.78	381.40	1.83	0.07	−69.28	1462.85

## Discussion

### Stress, income and Klotho

We found a significant positive association between α-klotho levels at 2 years and increased household income  possibly related to increased stress exposures in lower income households. In a previous study examining psychosocial factors in adults in relation to α-klotho levels, high-stress caregivers had lower serum α-klotho levels than low-stress ones [14]. Lower α-klotho levels were also significantly associated with depressive symptoms, a common maladaptive brain response to stress [14]. Other studies have shown that poverty increases stress responses not only in parents [15] but children [15] as well, affecting both physiological [16] and psychological [17] development even at very young ages. Efforts to alleviate poverty have been suggested to improve health outcomes in children [16]. In our LEAD cohort, mothers who had higher housheold incomes may have had children who experienced less chronic stress and therefore had higher α-klotho levels, than children in lower income households and higher exposures to stress.

### Sugar sweetened beverages

We found a positive association between SSB intake and higher α-klotho levels supporting a potential association between secreted α-klotho levels and phosphate in young, low-income children [18, 19]. Children at 24 months of age who reported drinking any SSBs showed significantly higher α-klotho levels (2029.78 ± 999.45 pg/mL) than those who did not (1402.78 ± 496.28 pg/mL) even when controlling for weight Z score at birth, leptin levels at two years, insulin at birth, child sex, maternal education and household income levels. Our findings suggest that dietary sources of phosphate as a food additive can influence this relationship [8]. Phosphate is commonly used as an additive in sugary drinks marketed to children and especially colas [8], commonly consumed by young children, particularly in higher risk low-income communities. Fifty-nine percent of our cohort was already consuming SSBs by 2 years of age.

 Phosphorous additives on food labels can be difficult to decipher  and, the amount of phosphorous in sugar sweetened beverages can range from < 1 mg to 175 mg per serving [20]. Even if parents in our study were avoiding giving their children carbonated sodas, the drinks they may have assumed were better alternatives contain high amounts of phosphorous per serving: Hawaiian Punch (175 mg), Fruit Works (63 mg-140), Aquafina Flavor (62 mg–85 mg), Tropicana Fruit Punch (93 mg) [20]. Even amounts of approximately 60 mg per serving serve as significant sources of dietary phosphates. The organic absorption rate of phosphates from meats, dairy, grains and beans is 40–60% [21] while the absorption rate  from inorganic phosphates in SSBs, processed meats and cheeses [15] can be as high as 100% [22].

The physiological regulation of phosphate is controlled by the FGF-23/α-klotho mechanism such that an increase in phosphate levels is associated with an increase in both FGF-23 [23]^24^ and α-klotho levels [1, 6]. An increase in phosphate levels signals the FGF-23 complex to trigger a cascade which reduces the number of sodium-dependent phosphate (NaPi) cotransporters, type-2a (NaPi-2a), on the brush border membrane of proximal tubules, thereby promoting renal phosphate excretion [24]. α-Klotho is required for FGF-23 to induce negative phosphate balance in this mechanism, demonstrated in previous studies where FGF-23 recombinant protein is unable to reduce serum phosphate levels in klotho deficient mice [24]. While α-klotho is a necessary co-receptor of phosphate regulation [25], α-klotho also exhibits effects independent of FGF-23 and parathyroid hormone (PTH), which regulate calcium ion homeostasis in the kidneys [23]. While previous evidence supports that high α-klotho levels have positive physiological effects [25] and benefits to cognition [26], the elevation of α-klotho levels via activation of the FGF-23 phosphate regulation pathway raises concern of potential long term harm.

As significant sources of inorganic phosphates which are readily absorbed, SSBs create an artificial increase in FGF-23. Elevated FGF-23 is associated with negative health consequences including inflammation [23] and is an early marker for chronic kidney disease [4]. It is not clear whether the positive effects of increased α-klotho levels would compensate for potential harm of chronically elevated FGF-23 levels. Our previous study found that insulin at birth was inversely correlated with cord blood α-klotho levels [11] .There may be a confounding relationship between α-klotho, SSBs and insulin. Controlling for insulin in our study strengthened the relationship between α-klotho and SSBs . Future studies should also assess FGF-23 in addition to α-klotho levels.

Any consumption of SSBs in children, may be positively associated with α-klotho levels supporting previous studies that high phosphate diets and FGF-23/ α-klotho mechanisms are related [9, 18]. The initial increase in α-klotho may be an immediate response in the short term to control phosphate levels [22] but could have lasting negative effects. High FGF-23 concentrations are linked to cardiovascular damage and inflammation [4]. Intake in early childhood could be particularly damaging as high dietary phosphates has been shown to be more detrimental at critical growth periods [7].

Dietary habits are known to be established early in life and previous evidence supports that diet quality tracks and declines from early childhood through adolesence [27]. SSB consumption in early childhood could track through adolescence [27] especially with increased exposure to advertising [28] leading to a lifetime of increased consumption [29].

### Limitations

Future studies should include additional metabolic and micronutrient serum indicators. We did not assess serum phosphate, FGF-23 or vitamin D levels in our participants. These serum markers would have provided a more comprehensive understanding of the relationship between dietary phosphate intake, including inorganic phosphate from SSBs, α-klotho and metabolic profile including Vitamin D status [1]. Further, future studies should account for all dietary sources of phosphate including those from other high phosphate foods (e.g. beans, lentils, pasta and rice) as well as intake of other source sources of inorganic phosphate from  fast foods, other processed foods and canned foods.

## Conclusions

The positive association between SSB intake and α-klotho levels at 2 years may reflect higher phosphate levels consistent with SSB intake in healthy children. The early intake of SSBs may establish potentially life-long habits of consumption. Poteintial associations between inorganic phosphates found in SSBs and α-klotho protein may result in increased inflammation,  suggesting that there may be a complex interplay between dietary intake and α-klotho protein levels. We additionally found evidence of a possible relationship between family stress or low socioeconomic status and lower serum α-klotho levels, similar to other published findings. The complex relationship between environmental exposures including psychosocial and dietary ones and child serum α-klotho levels necessitates further longitduinal study at different ages.

## Data Availability

Data is publicly available by written request and after review from Janet M Wojcicki (janet.wojcicki@ucsf.edu).

## Abbreviations

BMI: Body mass index
CI: Confidence interval
CV: Coefficient of variation
FGF-23: Fibroblast growth factor − 23
NaPi: Sodium-dependent phosphate
SSB: Sugar sweetened beverages
PTH: Parathyroid Hormone
WIC: Special Supplemental Program for Women, Infants and Children
